# The Role of the Gap Junction Protein Connexin in Adrenal Gland Tumorigenesis

**DOI:** 10.3390/ijms25105399

**Published:** 2024-05-15

**Authors:** Maja Mizdrak, Tina Ticinovic Kurir, Ivan Mizdrak, Marko Kumric, Mladen Krnic, Josko Bozic

**Affiliations:** 1 Department of Internal Medicine, University Hospital of Split, 21000 Split, Croatia; mmizdrak@mefst.hr (M.M.); tina.ticinovic.kurir@mefst.hr (T.T.K.);; 2 Department of Pathophysiology, University of Split School of Medicine, 21000 Split, Croatia; marko.kumric@mefst.hr; 3 Department of Otorhinolaryngology, Head and Neck Surgery, University of Split School of Medicine, 21000 Split, Croatia; imizdrak@kbsplit.hr; 4 Laboratory for Cardiometabolic Research, University of Split School of Medicine, 21000 Split, Croatia

**Keywords:** connexin, gap junction, intercellular communication, adrenal gland, adrenocortical cancer, pheochromocytoma

## Abstract

Gap junctions (GJs) are important in the regulation of cell growth, morphology, differentiation and migration. However, recently, more attention has been paid to their role in the pathogenesis of different diseases as well as tumorigenesis, invasion and metastases. The expression pattern and possible role of connexins (Cxs), as major GJ proteins, under both physiological and pathological conditions in the adrenal gland, were evaluated in this review. The databases Web of Science, PubMed and Scopus were searched. Studies were evaluated if they provided data regarding the connexin expression pattern in the adrenal gland, despite current knowledge of this topic not being widely investigated. Connexin expression in the adrenal gland differs according to different parts of the gland and depends on ACTH release. Cx43 is the most studied connexin expressed in the adrenal gland cortex. In addition, Cx26, Cx32 and Cx50 were also investigated in the human adrenal gland. Cx50 as the most widespread connexin, along with Cx26, Cx29, Cx32, Cx36 and Cx43, has been expressed in the adrenal medulla with distinct cellular distribution. Considerable effort has recently been directed toward connexins as therapeutically targeted molecules. At present, there exist several viable strategies in the development of potential connexin-based therapeutics. The differential and hormone-dependent distribution of gap junctions within adrenal glands, the relatively large gap junction within this gland and the increase in the gap junction size and number following hormonal treatment would indicate that gap junctions play a pivotal role in cell functioning in the adrenal gland.

## 1. Introduction to Connexins

Connexins (Cxs) are integral transmembrane proteins that form channels between neighboring cells and enable the bidirectional exchange of small hydrophilic molecules less than 1200 daltons, i.e., ions (Ca^2+^, K^+^ and bicarbonate), secondary messengers (inositol 3-phosphate and cAMP), reactive oxygen species, small molecules (glucose, amino acids, nucleotides, ATP and NAD+), peptides and microRNAs [1]. They participate in intercellular communication or communication between the intracellular and extracellular milieu [2]. There are 21 isoforms of connexins in humans, named by their molecular mass expressed in kilo Daltons ranging from Cx23 to Cx62 [3,4]. Alternatively, connexins are classified based on their sequence homology into five subfamilies (α, β, γ, δ and ε) and are named with the prefix—the GJ (gap junction)—while the consecutive number indicates the order of discovery [5]. Connexins are expressed in all tissues, except for skeletal muscles, erythrocytes and mature sperms [5]. They are synthesized in the endoplasmic reticulum, oligomerized into connexons in the Golgi apparatus and degraded in lysosomes and proteasomes [5,6]. The connexins are localized at the plasma membrane of human cells but also might appear inside cells, in mitochondria or in a soluble form and are rarely in exosomes [1]. They are the structural proteins of gap junctions placed in lipid rafts enriched with cholesterol and sphingolipids [7]. They consist of four transmembrane domains; two extracellular loops and one intracellular loop; and intracellular N- and C-terminal tails [1,5,8,9]. Six connexins form a hemichannel or connexon, and two connexons form gap junctions (GJs).

Connexins have a relatively short half-life of 1–5 h, implicating that communication via the gap junction, as a cornerstone of intercellular communication, is a permanent and dynamic process [9]. Connexins have roles in different physiological processes, i.e., angiogenesis, cell growth, cell cycle regulation, differentiation, wound healing and tissue homeostasis [10]. They accomplish tissue integration by transmitting electrical and chemical signals, maintaining cell polarity, sustaining differentiation and providing nutrients and oxygen to non-vascularized tissues, and these effects are based on their main role of substance exchange [1]. These findings are the foundation of further investigations on how to alter connexin expression and thus intercellular communication [11]. An altered connexin expression pattern is associated with the pathogenesis of neurodegenerative, cardiovascular, liver, lung and kidney diseases and, finally, tumorigenesis. Different connexins were studied in carcinogenesis, and among them, the most studied is Cx43 [1]. The aim of this study was to analyze the dual connexin roles as tumor suppressors and tumor promoters, especially emphasizing and making clear their role in adrenal gland pathophysiology.

## 2. Connexins—Tumor Suppressor or Tumor Promoter?

Gap junction intercellular communication (GJIC), as a regulator of tumor growth, was first studied in the 1960s in a seminal ex vivo study, in which authors have demonstrated the loss of electrical coupling in rat liver tumors [12]. Decades later, gap junctions and connexins have been constantly studied in carcinogenesis [13]. However, the accumulation of knowledge about this connection has shown an illusory contradictory relationship where connexins might be tumor suppressors or act pro-tumorigenically by promoting cancer cell growth, migration, invasiveness and, finally, metastases [2,13]. Their pro- or anti-tumorigenic behavior depends on their abundance, localization, tissue types, cancer stage and connexin isoforms, as well as their non-channel functions [1]. Connexins are involved in all phases of tumorigenesis through different mechanisms, firstly being GJIC mediators or independently of GJIC through their interactome or as hemichannels mediating autocrine and paracrine communication [12,13]. This complex involvement of connexins in cancer progression is even more complicated by the fact that their hemichannel function may overlap with other GJ-related proteins—the pannexins—which, to the contrary, do not form hemichannels [13]. Furthermore, due to the dysregulation of connexin expression, aberrant GJIC might be present in cancer. It occurs at the transcriptional, the post-transcriptional, the protein-synthesis or the post-translational level [12]. According to the data from the literature, connexins mostly act as tumor suppressors, i.e., Cx32 in malignant tumors of many sites, Cx26 and Cx43 in breast cancer and Cx43 in lung cancer and melanoma, as well as Cx37 and 40 in laryngeal squamous cell cancer [12,14,15,16]. Several connexin-knockout mouse models have supported this theory. Namely, Cx43-knockout mice more often develop lung cancer and Cx32-deficient mice have an increased incidence of liver tumors after exposure to carcinogens [1]. There is plenty of evidence that GJIC is critical for normal cell function and its loss promotes the malignant transformation of the cells [10]. Changes in the overall connexin abundance, but predominantly its loss, are associated with tumorigenesis under the action of various transforming factors, such as metabolic disorders, inflammation or bacterial infection; one example is non-alcoholic hepatosteatosis and the consequential downregulation of Cx32 with the final step—the development of hepatocellular carcinoma [1]. The cytoplasmic C-terminal tail domain of Cx43 may also act to suppress, as does the full protein. Connexins might also suppress tumorigenesis through channel-independent mechanisms as well as interact with other proteins or signaling pathways by affecting their cellular location, i.e., β-catenin for Cx43, or by changing the conformation of other proteins (i.e., the inactivation of c-Src) [12]. They can contribute to the epithelial–mesenchymal transition (EMT). For example, overexpression of Cx43 can promote the epithelial phenotype due to the inhibition of MAPK/ERK and Akt signaling, while Cx26 and Cx43 can reduce cell migration, increase cytokeratin 18 expression and decrease the vimentin level [17]. Connexins affect the production and activity of many cell cycle regulators, including p27Kip1, cyclin A, cyclin D1, cyclin D2, ERK1/2, Src and FGF1. Furthermore, secreted factors from connexin-overexpressing cells inhibit endothelial cell tubulogenesis and migration. However, when normal stromal communication is lost, tumor cells multiply and start to prevail in the microenvironment and thus connexins become a tumor promoter [1].

Some connexins act in a contrary way to that mentioned above and provoke both tumorigenic and metastatic potential as well as drug resistance. The examples from the literature are Cx26 in melanoma or Cxs30 and -43 in glial tumors [12,18,19]. This effect is manifested by a decrease in the connexin amount, their relocation, a change in the spatial expression pattern, i.e., being constantly expressed where they are only transiently expressed or de novo expression where they do not usually appear [1]. The loss of characteristic connexins or the overexpression of non-typical connexins in some tissues causes the upregulation of oncogenes and stemness markers [1]. In normal breast development, Cx32 is expressed only during lactation and its overexpression appears in metastatic breast cancer as well as expression of Cx46, which is not present in normal breast tissue and appears in the early stages of breast cancer [1]. Furthermore, Cx26 and Cx43 have a role in the early steps in the metastasizing process in animal models of brain metastases occurrence [12]. Their depletion in melanoma and breast cancer inhibits brain colonization by blocking tumor cell extravasation [12]. Glioma tumor cells directly form a gap junction with the surrounding glial cells, becoming more invasive and treatment resistant [20]. Brain metastatic cancer cells establish gap junctions with astrocytes to promote tumor growth and chemoresistance [12]. Connexins can form heterologous gap junctions between tumor cells and endothelial cells to facilitate intravasation and extravasation [12]. Finally, Saito-Katsuragi et al. have found that Cx26 has a role in melanoma acquiring malignant potential and promoting intercellular communication with neighboring endothelial cells [20]. Their study has shown that Cx26 expression was not found in some secondary lesions, despite being established in the primary tumor. These results suggest that although Cx26 might be crucial during the metastatic process, it might be nonessential for the progression of tumor cells [20]. To make this story even more complex, there is evidence that in some glioma subtypes there is an opposite connexin role, for example, Cx43 has both a pro-angiogenic and an anti-angiogenic role in the same cancer, for which the mechanism was not clear. Furthermore, the homocellular GJ between glioma cells can inhibit metastasis, while heterocellular GJs formed between glioma cells and astrocytes can promote metastasis in a GJIC-dependent manner. In conclusion, the major question of the connexins’ role as tumor suppressors or tumor promoters still remains unanswered and is a matter for future research, despite all that has by now been discovered.

## 3. Gap Junction-Mediated Intercellular Communication in the Adrenal Gland

**Figure 1:**
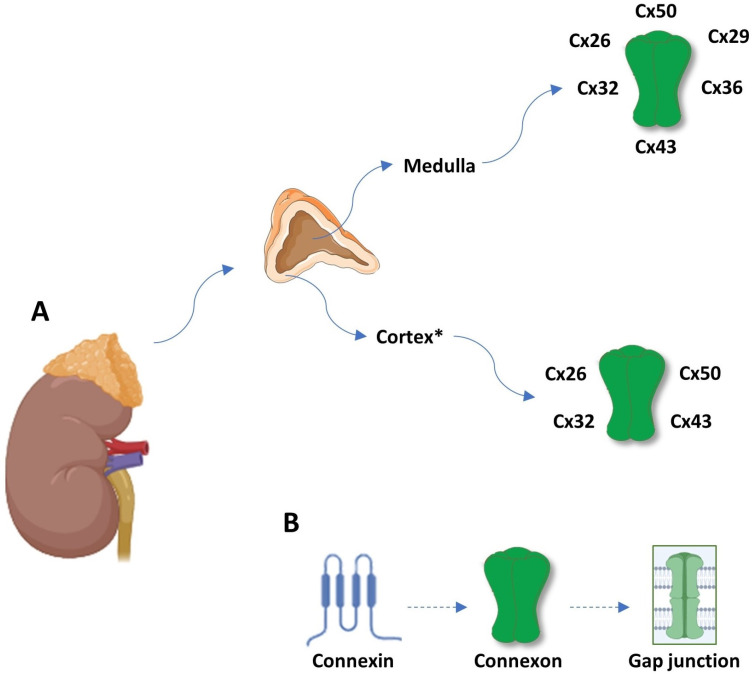
(**A**) Connexin expression pattern in adrenal gland dependent of zonation, being the highest in zona reticularis. (**B**) Schematic review of gap junction structure; a connexin is a protein with four transmembrane domains, two extracellular loops and one intracellular loop, and intracellular N- and C-terminal tails. Six connexins form a hemichannel or connexon, and two connexons form a gap junction. * ZR > ZF >> ZG. Abbreviations: Cx—connexin; ZG—zona glomerulosa; ZF—zona fasciculate; and ZR—zona reticularis.

The adrenal gland has a cortex and medulla, two completely different tissues in both function and structure that are still interdependent. The chromaffin cells of the medulla have a neuroendocrine function, while the epithelial cells of the cortex have a function in the endocrine metabolism [21]. Interestingly, the cells from the adrenal cortex may display neuroendocrine characteristics while the GJ can contribute to the functional interdependence between them [22]. The adrenal cortex consists of the zona glomerulosa (ZG), zona fasciculata (ZF) and zona reticularis (ZR). The inner zones secrete glucocorticoids and adrenal androgens in response to ACTH (adrenocorticotropin). The zona glomerulosa produces mineralocorticoids (aldosterone) in response to electrolyte and water disturbances. It has been more than 50 years since the first reported analysis of gap junctions in the adrenal gland [23]. It was suggested that the distribution of gap junctions was diverse with larger and more abundant GJs in the ZF and ZR compared to the ZG [24,25]. It means that more gap junctions were present in the more slowly proliferating inner cortical areas (being higher in the ZR compared to the ZF) with a greater capacity to communicate growth-inhibitory signals, and obscure connexin expression was detected in the more rapidly dividing zona glomerulosa that would result in more cell proliferation in this zone [25,26]. Cx43 is the most studied connexin expressed in the adrenal gland [22,27,28]. In addition, Cx26, Cx32 and Cx50 were also investigated in the human adrenal gland (Figure 1) [22,27,28]. The Cx43 gap junctions in the adrenal cortex appear as small puncta or longer plaques on the cell surface between contacting cells. According to the data from the present literature, Cx50, as the most widespread connexin along with Cx26, Cx29, Cx32, Cx36 and Cx43, has been expressed in the adrenal medulla with distinct cellular distribution [29].

Connexin expression in the adrenal gland differs according to different parts of the gland and depends on ACTH release [30]. Experimental studies have shown that ACTH increases connexin expression, namely, Cx43, whose distribution correlates with the proliferation rate and zonal response to ACTH [24,26]. The higher connexin expression after treatment with ACTH supports the idea that gap junctions are hormonally regulated and strongly correlated to increased steroidogenesis and decreased cell proliferation. A connection between ACTH and connexins as the major GJ protein was first studied in early cell culture experiments of ACTH-sensitive and non-sensitive Y-1 adrenal cortical cells [31]. The results have shown that an ACTH-sensitive clone of Y-1 cells owned GJs whose size increased after ACTH was added [31]. The importance of this association was established through multimodal gap junction inhibition. For example, the GJ inhibition by treatment with 18 alpha or beta-glycyrrhetinic acid decreased the ACTH-induced steroidogenesis and increased cell proliferation in experimental studies of bovine adrenal cells [32]. Gap junctions have important roles in numerous physiological processes in the adrenal gland, including steroidogenesis and decreasing proliferation [21]. ACTH binds to melanocortin 2 receptors on the zona fasciculate cells, which show restricted tissue specificity. These receptors are the part of the type 1 G protein-coupled receptor superfamily consisting of the melanocortin 2 receptor and the melanocortin 2 receptor accessory protein [33]. ACTH binding potentiates hormone synthesis in an acute and a chronic manner through the activation of the adenylyl cyclase, cyclic adenosine monophosphate production, protein kinase A activation and, finally, phosphorylation of specific nuclear factors [34]. This activation in one cell does not affect steroidogenesis in another cell if GJIC is decreased causing the loss of intercellular communication [30]. In experimental studies manifested with hypophysectomy and low ACTH levels, the atrophy of the adrenal cortex, especially the ZF and ZR, and the consequential connexin gap junction protein loss were noticed [35]. In contrast to the adrenal cortex, in the medulla, GJIC through connexins contributes to the catecholamine release, which is a key event in response to stressors [29]. A rise in cytosolic calcium is a major step in the triggering of catecholamine secretion [36].

## 4. Gap Junction Intercellular Communication in Adrenal Gland Tumorigenesis

Adrenal tumors have a prevalence of 3% to 10% in the adult population, being mostly non-functional adenomas of benign character [37]. Oppositely, adrenal malignant tumors are rare tumors with a poor prognosis [38]. The adrenocortical carcinoma and pheochromocytomas have an estimated incidence of 0.7–2.0 cases/million and 2–8 cases/million, respectively [39,40]. Although most pheochromocytomas are benign, the metastatic disease may develop in 15–17% [40]. For more than several decades, the gap junction’s role in the regulation of carcinogenesis, and in the adrenal gland, was speculated via the loss of the GJIC and alteration of the connexin expression pattern. As well as the gap junction’s ambiguous role being emphasized in the other tumor types, the same was noticed in adrenal gland studies, including the contradictory loss of gap junctions and the increase in connexin expression in some malignant lesions [41,42,43,44], Table 1 and Table 2.

A change in connexin expression might contribute to the malignant transition of the chromaffin cells of the adrenal medulla. Experimental studies in mice have shown that deletion of the Cx32 gene caused the development of non-secreting tumors of the adrenal medulla with a poor outcome [67]. In the other experimental study on rat tumor cell lines, only Cx36 expression has been noticed [73]. Willenberg and colleagues have evaluated the expression pattern of different connexins in the chromaffin cells of 10 normal human adrenal glands and 23 pheochromocytomas as a first study to evaluate expression of connexins in human malignant pheochromocytomas [27]. Their results have confirmed predominant Cx50 expression in the adrenal medulla and Cx43 expression in the adrenal cortex, while Cx43 was present and Cx50 was low in malignant pheochromocytomas in comparison to normal adrenal medulla and benign pheochromocytomas [27]. Cx26 and Cx32 were distributed inhomogeneously with no emphasis of expression in the types of tissues analyzed [27]. Despite promising results, authors have concluded that immunohistochemical staining of connexin expression could not differentiate benign from malignant pheochromocytomas. Stimulus–secretion coupling in the adrenal medulla is probably modified in tumor cells and consistent with the change in GJIC [29]. Contrary to the adrenal medulla, most tumors of the adrenal cortex develop in the zona fasciculata [24,25,26,28]. Results from the experimental studies have confirmed that in a normal adrenal gland, the GJ number is higher than in benign adrenal tumors and decreases even more in malignant adrenocortical carcinomas [28,74]. Specifically, Cx43 is a major connexin of the normal adrenal cortex whose expression diminishes with the higher malignant potential, and this fall means the loss of GJIC is linked to the tumor stage, differentiation and progression [21,28]. The gap junction role in the adrenal gland was further studied in a tumor cell line SW-13. After dibutyryl-cAMP (a cell-permeable PKA activator) treatment, there was increase in the gap junction number and a decrease in the size and cell proliferation, implicating GJ turnover [75]. These findings support the idea that the loss of GJIC as a result of alterations in gap junction trafficking and increased degradation in early cancer stages and the decrease in gap junction protein synthesis in later stages might cause the loss of growth regulation [24]. In benign adrenocortical adenomas, the number of gap junctions was significantly reduced in comparison to a normal adrenal gland where the ZF has the highest number of gap junctions. However, despite observed differences, this still lacks differentiated tumor behavior and has obscure clinical significance [45,46,47,48,49,50]. Furthermore, somatic mutations of CADM1 (neuronal cell adhesion gene) cause reversible hypertension and reveal a role for gap junction-mediated intercellular communication in suppressing physiological aldosterone production [45,46,47,48,49,50]. In experimental studies, CADM1 knockdown or mutation-inhibited gap junction-permeable dye transfer are considered.

## 5. Dysregulation of Connexins in Cancer: Therapeutic Opportunities and Future Perspectives

**Figure 2:**
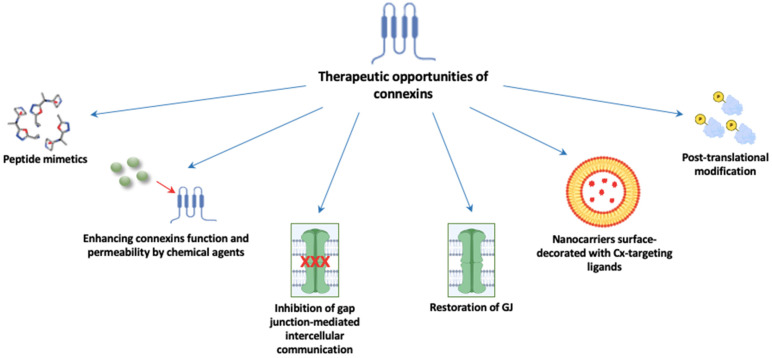
Possible implications of gap junctions as therapeutically targeted molecules. Abbreviations: GJ: gap junction; Cx: connexin.

Connexin-associated regulation is complex and includes the modulation of gap junction intercellular communication, hemichannel signaling and gap junction-independent pathways [12]. Whether it occurs in a GJIC-dependent or GJIC-independent way, the overall problem remains unsolved regarding the many contradictory data considering connexins as tumor suppressors or tumor promoters. The regulation of connexin expression includes different stages, from transcription to degradation, each providing an opportunity to modulate the connexin levels and functions in the cell [76]. In adrenal gland pathology, the connexin role is even more ambiguous due to the obscure data about their expression pattern and roles in the adrenal gland under both physiological and pathological conditions. Further, genetic and biochemical studies should clarify many of these issues. Due to all that is mentioned above, especially their potential role in cancer metastasis, connexins are amenable to become targeted therapeutic interventions in different cancers, especially in malignant adrenocortical carcinomas where therapeutic possibilities are unfortunately still limited. The permeability of specifically Cx43, as the most investigated connexin family member, to small molecules and macromolecules makes it a highly attractive target for delivering drugs directly into the cytoplasm [77]. Possible therapeutic approaches include connexin mimicking, GJIC inhibition, chemical agents capable of enhancing connexin function and permeability and nanocarriers surface-decorated with Cx-targeting ligands (Figure 2) [77].

The main aim of this potential therapeutical approach is gap junction and connexin restoration as anti-tumor agents in early cancer stages or inhibition of their pro-tumor role in advanced cancer stages [78]. Due to their paradoxical tumor-suppressing and tumor-promoting roles, these attempts become even more complex. There are plenty of examples of chemotherapy toxicity and drug resistance in clinical practice. One possible therapeutic connexin utility is the Cx43 gene transfer into a targeted cell and the expression elevation to promote cell sensitivity to oncological treatment shown in different tumors, such as prostate, colorectal and breast cancers [79]. Cx43 expression promotes cell permeability to chemotherapeutics as another possible therapeutic benefit, i.e., Cx43 upregulation enhances permeability to paclitaxel and doxorubicin in glioblastoma [80]. Furthermore, therapeutic peptide alpha Connexin Carboxy-Terminus 1 stimulates the Cx43 increased cell permeability to cytotoxic agents in breast cancer cells lines [10]. The combination of Cx32 and vinblastine helped with vinblastine sensitivity of clear cell renal carcinoma to the therapy [76]. Connexins can also promote “death signals” spreading and thus improve therapy cytotoxicity [81,82,83].

Furthermore, gap junction restoration with a therapeutic peptide ACT1 might help to enhance Cx43 function and impair the proliferation or survival of breast cancer cells [84]. A combination of ACT1 with standard oncological treatment potentiates the effects of these drugs on hormonal-positive breast cancer [84]. However, to the contrary, as opposed to that mentioned above, an increase in connexin expression might have the opposite effect. The most studied example is in malignant melanoma where overexpression of Cx26 was linked to the spontaneous metastasis of mouse BL6 melanoma cells [84]. Oleamide, the amide derived from the fatty acid oleic acid, was a selective inhibitor of Cx26-mediated intercellular communication and this therapy was associated with a potent anti-metastatic effect [76]. Another therapeutic option to inhibit gap junction function are connexin-targeting antibodies. Experimental studies on a rat glioblastoma model have shown that the monoclonal antibody that targets the EL-2 loop of Cx43 causes major tumor reduction and prolonged survival. However, this therapy associated with radiotherapy further decreased tumor development [85]. Another example of the Cx43 connection with chemotherapy resistance is the upregulation of Cx43 contributing to a decreased glioblastoma response to temozolomide [77]. Overexpression of Cx43 promotes chemoresistance via mitochondrial apoptosis signaling [84]. Blocking gap junctions can prevent GJIC and consequently promotes apoptosis [77]. In experimental studies, a Cx43 GJ inhibitor carbenoxolone was added to the tumor necrosis factor-related apoptosis-inducing ligand and these combined therapies ameliorated glioma cell apoptosis and thereby the survival rate [86]. Carbenoxolone also reduced nerve stimulation-evoked catecholamine release via GJ reduction [87]. The main strategy in GJIC-based therapies relied on the “bystander effect” (BE), a mechanism where a cytotoxic molecule or signal is transferred from targeted cells to adjacent cells and does not require the initial treatments to reach all tumor cells [76]. It happens through GJIC, and changes in the connexin levels translate into changes in the BE [76]. Finally, novel tumor treatments include cisplatin-loaded nanogels conjugated to monoclonal antibodies targeting Cx43, which inhibited tumor cell growth and increased survival in malignant glioma [88].

Patients’ own immunity encouragement is another option in the modern era of tumor treatment via gap junction-mediated cell coupling and antigen transport [78]. Connexins, through their main intercellular task, the exchange of small molecules and ions, might also ameliorate reactive oxygen and nitrogen species transfer and thereby cause cell death via oxidative stress, which damages membrane DNA, lipids and proteins [89]. Therefore, non-thermal plasma therapy (NTP) has potential on the function of GJs via oxidative stress reduction [90]. NTP mediates reactive oxygen species levels in an intensity- and time-dependent manner, for example, high levels of ROS mediated by NTP cause oxidative stress damage and cell death, while being the opposite in physiological levels [78,91]. In experimental studies of malignant melanoma, treatment with NTP increased gene expression in the apoptotic pathway and oxidative stress, while it decreased genes related to cell migration [78,91]. Cx26, whose role in melanoma was explained above, was upregulated following treatment with NTP and it maintained functionality during the onset of treatment [78]. Authors have concluded that gap junctions both increase the efficacy of NTP and perpetuate a positive feedback mechanism of gap junction expression and tumoricidal activity [78].

It is important to mention the GJIC-independent manner in tumor regulation. Cx43 can decrease breast cancer metastasizing through epithelial phenotype enhancing, i.e., N-cadherin expression and apoptosis promoting [21]. There is also evidence that connexin hemichannels can be active in single plasma membranes and trigger intercellular signaling without being incorporated into gap junctions [84]. Connexin phosphorylation plays an important role in the regulation of their levels and functions. Phosphorylation by the kinase Src is a major regulatory event in the life of connexins, either directly or via signaling intermediates [92]. This effect has been shown to result in drug resistance [84]. Finally, microRNAs, small non-coding RNAs with a broad range of functions essential in gene expression regulation, also have a role in connexin expression regulation. miR-1 and miR-206 have been shown to regulate/inhibit Cx43 [93].

Naus et al. have summarized the results of translational research of the potential connexin therapeutical implication in real-life treatment. The first of them was the hemichannel-blocking peptide RRNYRRNY, which blocked mitochondrial Cx43 hemichannels and mitochondrial calcium ion uptake [94]. A 25aa peptide (αCT1) with a compact 2-domain design binding domain of Cx43 (Granexin^®^, Mount Pleasant, SC, USA, 29464) was developed for acute and chronic cutaneous wounds. Modafinil is a standard of care used in narcolepsy and it enhances astrocyte GJIC. CoDa has three connexin channel modulators in clinical development, in the first instance for chronic skin wounds and ocular disease. These include an antisense oligodeoxynucleotide (Nexagon^®^, Tampa, FL, USA, 33606), which transiently downregulates Cx43 protein expression; an extracellular-acting Cx43 peptidomimetic that can be delivered locally or systemically (Peptagon™, Tampa, FL, USA); and a small molecule for systemic or oral delivery (HCB1019) [94]. The trial whose results we are awaiting is investigating Meclofenamate, which blocks gap junction-mediated intercellular cytosolic traffic in subjects with recurrent or progressive brain metastasis from a primary solid tumor [12]. Further research has shown that the inhibition of intercellular cytosolic traffic via gap junctions reinforces lomustine-induced toxicity in glioblastoma independent of the MGMT promoter’s methylation status [95]. Further studies are needed to elucidate the implication of connexin-targeted therapy in clinical practice, especially concerning their dual role. For example, would enhancing connexin expression in a primary tumor run a risk of more efficient metastatic spread or growth or the opposite?

In summary, with advanced understanding of the mechanism of connexins and gap junctions in different malignant diseases and metastasizing, drugs targeting connexins in cancer cells in the next phase of cancer therapy are edging closer to becoming a reality. Further research is needed both in vivo and in vitro to elucidate the expression pattern of each connexin in different tissues, including the adrenal gland, as well as to clarify its pro-tumorigenic or tumor-suppressor role and, finally, the potential therapeutic target.

## Figures and Tables

**Table 1:** Systematic review of connexin expression pattern in different benign adrenal masses with correlation to other organ sites.

	Connexin Expression Pattern in the Adrenal Gland	Connexin Expression Pattern of Specific Lesion of the Other Site	References
Adenoma	- Normal: the ZF has the highest number of gap junctions - In benign adrenocortical adenomas, the number of gap junctions was significantly reduced → obscure clinical significance - CADM1 (neuronal cell adhesion gene) knockdown or mutation inhibited the gap junction - Permeable dye transfer	- The overexpression of Cx43 decreases cell growth and induces apoptosis in pituitary tumor cell lines - Cx43—an additional factor indicative of the malignant potential of bowel cancer, while there is no difference in adenomas - The treatment with urethane was associated with the downregulation of Cx26, 32 and 46 expressions in urethane-induced mouse lung adenomas - Cx43 is overexpressed in Apc/Min (multiple intestinal neoplasia) +/− mice adenomas and colocalises with COX-2 in myofibroblasts	[28,45,46,47,48,49,50]
Amyloidosis	Unknown	- The activation of astrocytes, which are characterized by a high level of intercellular communication mediated by Cx43 and Cx30, surrounding amyloid plaques, is a hallmark of Alzheimer disease	[51,52,53,54,55,56]
Congenital adrenal hyperplasia	Unknown	-	-
Ganglioneuroma	-	Unknown	-
Granuloma	Unknown	- Granuloma cells express Cx43 in hepatic granulomas induced by Schistosoma mansoni eggs - Cx43 expression was reduced in Cx43 (+/−) mice	[57]
Hamartoma	Unknown	- Increased expression of Cx36 and Cx43 in human hypothalamic hamartoma compared with normal human tissue - Irregular Cx43 expression was observed in a rare cardiac hamartoma containing adipose tissue in the crista terminalis	[58,59]
Hemangioma	Unknown	- In comparison with angiosarcoma, the Cx43 expression in hemangioma was lower, suggesting that Cx43 may play a role in the development of malignant vascular tumors - Mice lacking Cx37 and Cx40, expressed in vascular endothelium, die perinatally with pronounced vascular abnormalities	[60,61]
Lipoma	Unknown	Unknown	-
Myelolipoma	Unknown	Unknown	-
Neurofibroma	Unknown	- Equal Cx43 expression in normal control and neurofibromatosis 1 keratinocytes - Cx43 was relatively evenly distributed in NF1 cells and did not form typical gap-junctional plaques between keratinocytes	[62]
Pheochromocytoma	- Immunohistological Cx expression does not still allow for differentiation of benign from malignant pheochromocytomas - Almost no immunoreactivity for Cx43, high Cx32 and low Cx50 expression in pheochromocytomas	-	[27]

**Table 2:** Systematic review of connexin expression pattern in different malignant adrenal masses with correlation to other organ sites.

	Connexin Expression Pattern in the Adrenal Gland	Connexin Expression Pattern of Specific Lesion of the Other Site	References
Adrenocortical carcinoma	- Normal: the ZF has the highest number of gap junctions - ACC: the lowest number of GJs	-	[27,28]
Angiosarcoma	Unknown	- Strong Cx43 staining in angiosarcoma	[60]
Leiomyosarcoma	Unknown	Unknown	-
Malignant schwannoma	Unknown	- Expression of Cx32 and 43 but not Cx26, 37, 40, 45 and 46 in schwannomas	[63,64]
Malignant primary melanoma of adrenal gland	Unknown	- Melanocytes express Cx43 and Cx46 and lower Cx26 and Cx30.2 - In melanocytic tumor tissues: the loss of Cx43, the fall of the cell membrane and elevated paranuclear Cx32 with moderately increased cytoplasmic Cx26 and paranuclear Cx30.2 during tumor progression - Significant downregulation of Cx31.1 expression in metastatic melanoma lesions	[65,66]
Malignant pheochromocytoma	- Cx26 was not present in normal chromaffin cells, and in pheochromocytomas, it was at a noticable level - Comparable to the normal adrenal cortex without a difference between benign and malignant pheochromocytomas - There were low Cx32 and Cx50 expressions in malignant pheochromocytomas - Cx43 was present within the malignant pheochromocytomas	-	[27,67]
Neuroblastoma	Unknown	- Cx26 is a tumor suppressor in gap junction-deficient mouse neuroblastoma cells - Exposure to dibutyryl-cyclic AMP causes the upregulation of Cx43 expression and phosphorylation in neuroblastoma cell lines - Cx43 is aberrantly located in IMR-32 cells (highly malignant human neuroblastoma cell line) emphasizing that these cells are incapable of gap junction-mediated intercellular communication	[68,69,70,71,72]
